# Mechanical properties and failure characteristics of geocells: A comparative experimental study

**DOI:** 10.1371/journal.pone.0351237

**Published:** 2026-10-23

**Authors:** Yuefeng Zhang, Da Zhang, Liang Wang, Wei Pang, Junxia Ding

**Affiliations:** 1 Jinshan Branch, Cangzhou Transportation Development (Group) Co., Ltd., Cangzhou, Hebei, China; 2 School of Traffic and Transportation, Shijiazhuang Tiedao University, Shijiazhuang, Hebei, China; 3 School of Civil Engineering, Shijiazhuang Tiedao University, Shijiazhuang, Hebei, China; Jazan University College of Engineering, SAUDI ARABIA

## Abstract

The tensile performance of geocells depends on the mechanical responses of both cell walls and junctions. Three commercial geocells, liquation-welded polypropylene (LW), injection-molded polyester (IM), and ultrasonic-welded high-density polyethylene (UW), were examined through tensile testing, macroscopic observation, and scanning electron microscopy of cell-wall fracture surfaces. Five configurations were used: cell-wall tension, junction-inclusive tension, and junction seam, shear, and split tests. The cell-wall peak strengths of LW, IM, and UW were 143.03 ± 4.43, 185.61 ± 7.96, and 39.42 ± 2.41 kN/m, respectively. Including a junction changed the strengths by +28.99%, −59.79%, and −23.76%. The LW and IM cell walls lost load rapidly after the peak. UW exhibited necking and extended post-yield deformation. SEM revealed microfibrillar bundles in LW, a smooth layered surface in IM, and dense drawn microfibrils in UW. Under junction-inclusive tension, LW tore near the raised-tab edge, IM cracked through the ligaments between the holes, and UW fractured at a weld-wall boundary. LW provided the highest seam and shear strengths of 66.16 ± 4.20 and 146.02 ± 8.46 kN/m. IM provided the highest split strength of 158.55 ± 10.34 kN/m. UW showed lower junction strengths but reached 343.09 ± 13.72% rupture strain under shear. Under seam and shear loading, LW failed near the tab-wall transition, while IM fractured from hole edges. UW failed within the weld region under seam loading and at a weld-wall boundary under shear loading. Under split loading, failure occurred at the heat-fused interface in LW, the molded body in IM, and the central weld in UW.

## 1 Introduction

Geocells are three-dimensional cellular geosynthetics formed by connecting polymer cell walls at regularly spaced junctions [1]. After a geocell is expanded and filled, outward movement of the infill mobilizes tensile resistance in the surrounding cell walls. This resistance confines the infill, redistributes applied stress, limits lateral deformation, and reduces settlement [2–4]. These functions have supported the use of geocells in road foundations [5–7] and railway engineering [8–10]. Their engineering performance relies on the cellular load-transfer network formed by the cell walls and junctions.

Studies of geocell-reinforced soil have examined the effects of cell geometry, wall stiffness, infill properties, and loading conditions on bearing response and deformation [11–14]. This work has clarified how confinement develops at the scale of the reinforced-soil system. Within a geocell, tensile force mobilized in one cell wall must pass through a junction before it can be redistributed to adjacent cells. The cell wall provides tensile resistance, while the junction transfers that resistance through the cellular network. Their mechanical responses therefore jointly influence the continuity of load transfer.

Component tests have provided important information on these two parts of the load-transfer path. Liu et al. examined HDPE cell walls and welded junctions under tensile, seam, shear, peeling, and split loading and reported loading-dependent strength and failure patterns [15]. Studies of polymer-blend and HDPE cell walls further showed that polymer composition, manufacturing history, and loading direction influence tensile strength and deformation [16, 17]. Tests on commercial connection forms also showed that junction construction affects deformation and failure position [18]. These studies indicate that the mechanical response of a geocell is related to both cell-wall properties and load transfer through the junction region.

Subsequent studies extended this understanding by examining pocket geometry [19], time-dependent deformation of geocell walls [20, 21], geocell–soil interaction [22], and biaxial response from individual strips to complete cells [23]. These studies extend current knowledge at the material, component, and reinforced-soil scales. However, component-level studies have mainly examined a single geocell type or a limited set of loading configurations. The cell-wall response, the change caused by placing a junction in the tensile load path, and junction resistance under seam, shear, and split loading have therefore rarely been compared for several commercial geocell types within one test program.

This study compares three commercial geocell types: liquation-welded polypropylene (LW), injection-molded polyester (IM), and ultrasonic-welded high-density polyethylene (UW) geocells. The experimental program includes cell-wall tension, junction-inclusive cell-wall tension, and junction seam, shear, and split tests. Strength and deformation responses, macroscopic failure development, and final failure positions are examined under each loading configuration. SEM observations of cell-wall tensile fracture surfaces are used to characterize the fracture morphology of the three materials. This experimental design provides a systematic comparison of the tensile response, junction resistance, and failure characteristics of the three geocell types under different load-transfer conditions.

## 2 Materials and methods

### 2.1 Materials

Three commercial geocell products were investigated: liquation-welded (LW), injection-molded (IM), and ultrasonic-welded (UW) geocells, as shown in Figs 1–3. The IM and UW geocells are established commercial products used in infrastructure engineering in China. They are manufactured by perforation-based injection molding and ultrasonic welding, respectively. The LW geocell is a recently developed product with integral raised tabs joined by heat fusion. These products were selected to compare the tensile responses and failure characteristics of the LW geocell with those of the established IM and UW geocells. The material composition, cell-wall manufacturing processes, geometric dimensions, and junction characteristics of the investigated geocells are summarized in Table 1.

**Table 1:** Specifications of the investigated geocells.

Specification	Liquation-welded geocell	Injection-molded geocell	Ultrasonic-welded geocell
Base polymer	polypropylene (PP)	polyethylene terephthalate (PET)	high-density polyethylene (HDPE)
Cell-wall manufacturing process	Stretching	Stretching	Extrusion
Cell-wall height (mm)	43.4	50.0	50.0
Cell-wall thickness (mm)	0.5	0.6	1.0
Junction configuration	Integral raised tabs joined by heat fusion	Injection-molded body formed through pre-formed holes	Overlapped strips joined by ultrasonic welding
Junction size (mm) Width × Thickness × Height	14.0 × 13.6 × 50.0	20.0 × 10.0 × 65.0	10.0 × 2.0 × 50.0
Junction spacing (mm)	400.0	400.0	400.0

**Fig 1:**
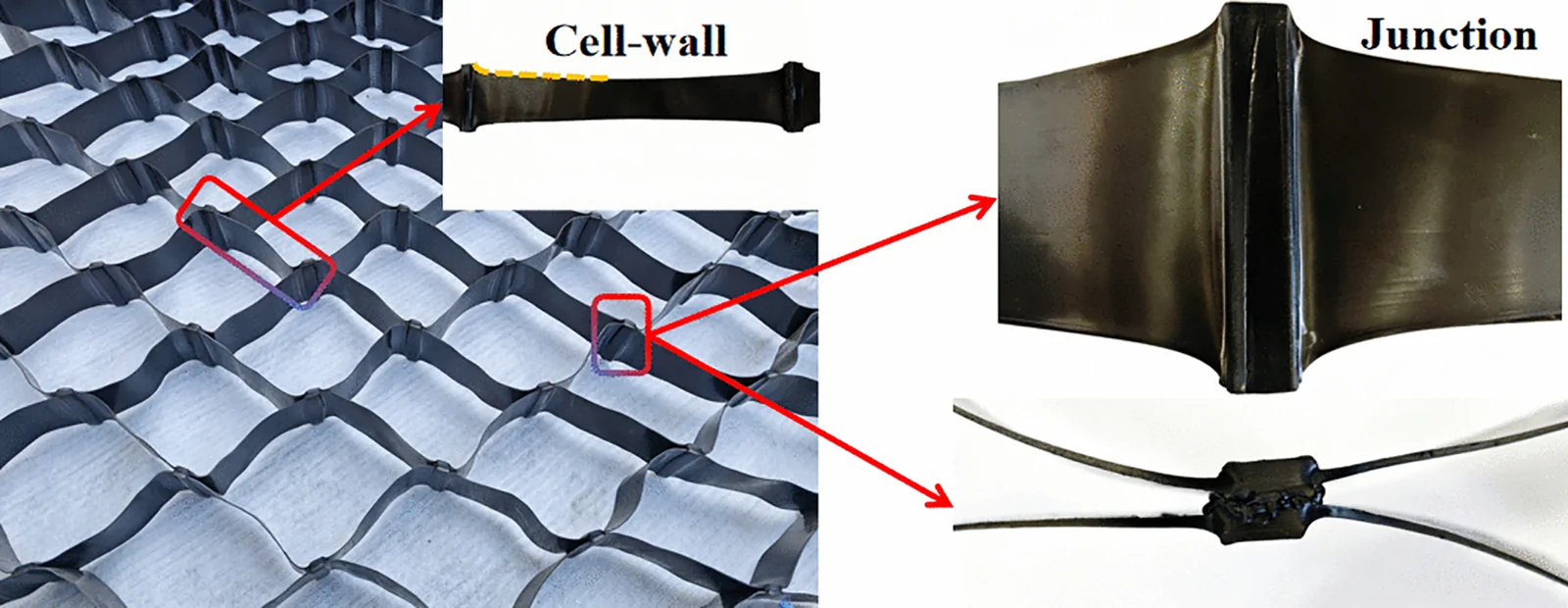
Liquation-welded geocell and integrated raised-tab junction.

**Fig 2:**
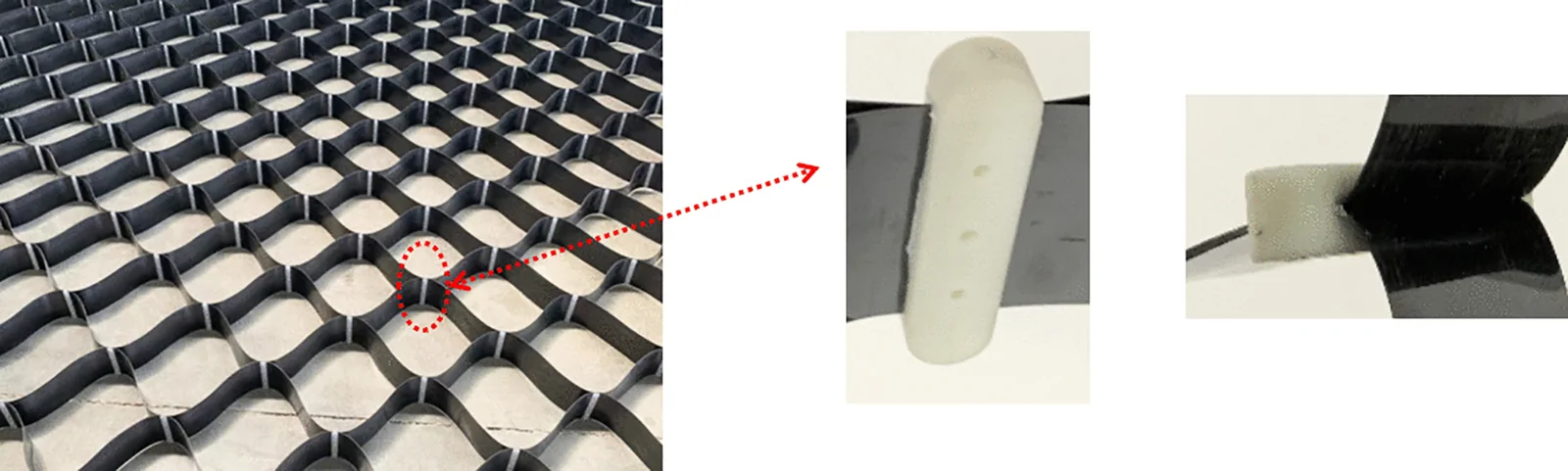
Injection-molded geocell and molded junction body.

**Fig 3:**
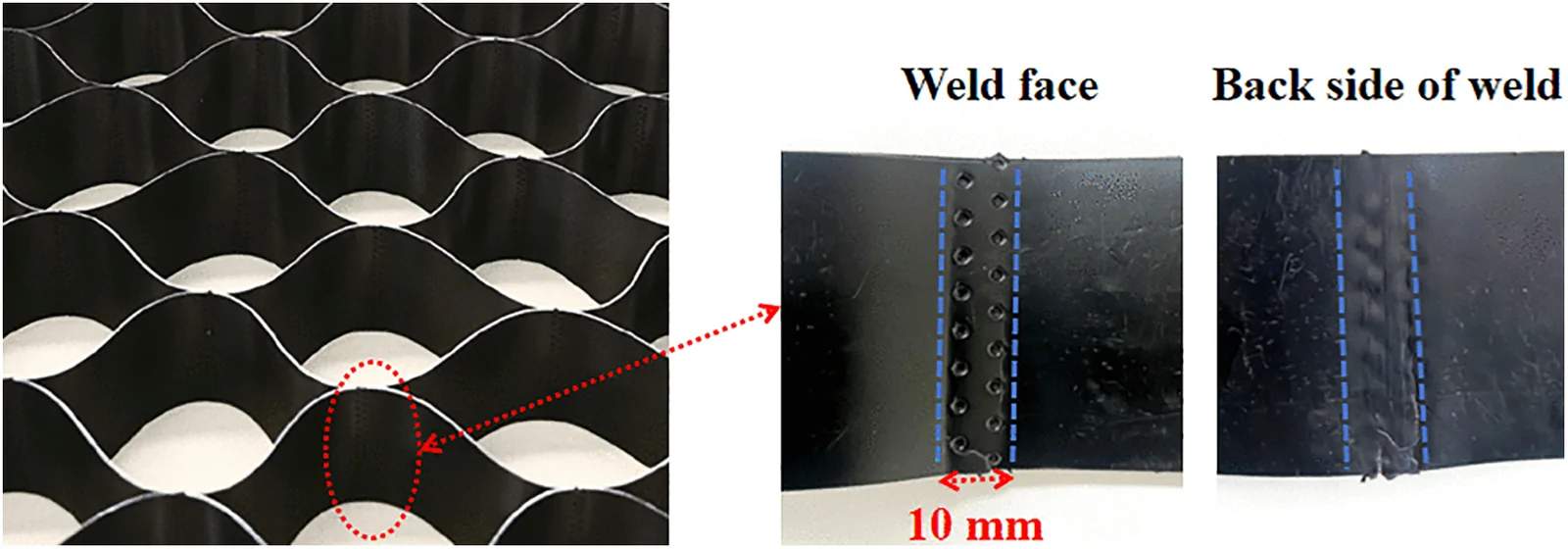
Ultrasonic-welded geocell and weld region.

#### 2.1.1 Liquation-welded geocells.

For the LW product, the polymer is formed in a customized die to produce continuous strips with integral protruding tabs. The tabs are held while the wall segments between adjacent tabs are stretched, creating the curved strip profile. Paired tabs from adjacent strips are then heat-fused to form the junction. The junction is therefore thicker and higher than the wall segment between junctions, and its formation does not require holes to be cut through the load-bearing wall.

#### 2.1.2 Injection-molded geocells.

For the IM product, holes are formed at the specified junction positions in stretched PET strips. Adjacent strips are aligned and fixed, and polymer is injected through the holes in a customized mold to create the junction body.

#### 2.1.3 Ultrasonic-welded geocells.

For the UW product, uniform HDPE strips are produced by extrusion. Adjacent strips are overlapped at the junction positions and joined by ultrasonic welding. During welding, high-frequency vibration is applied under clamping pressure. Localized heating softens the contact interface and consolidates the overlapping strips into a narrow weld region.

### 2.2 Test apparatus

Fig 4 shows the DW1210 universal testing machine used for all tensile tests. The machine uses constant-rate-of-extension control and records load, crosshead displacement, and time. Its measurement capacity and accuracy are listed in Table 2.

**Table 2:** Technical specifications of the geosynthetic tensile testing apparatus.

Specification	Value
Load measurement range (kN)	0–100
Load accuracy	±0.5% FS
Maximum tensile stroke (mm)	1100
Deformation measurement accuracy	±0.5% FS
Crosshead speed range (mm/min)	0–300

**Fig 4:**
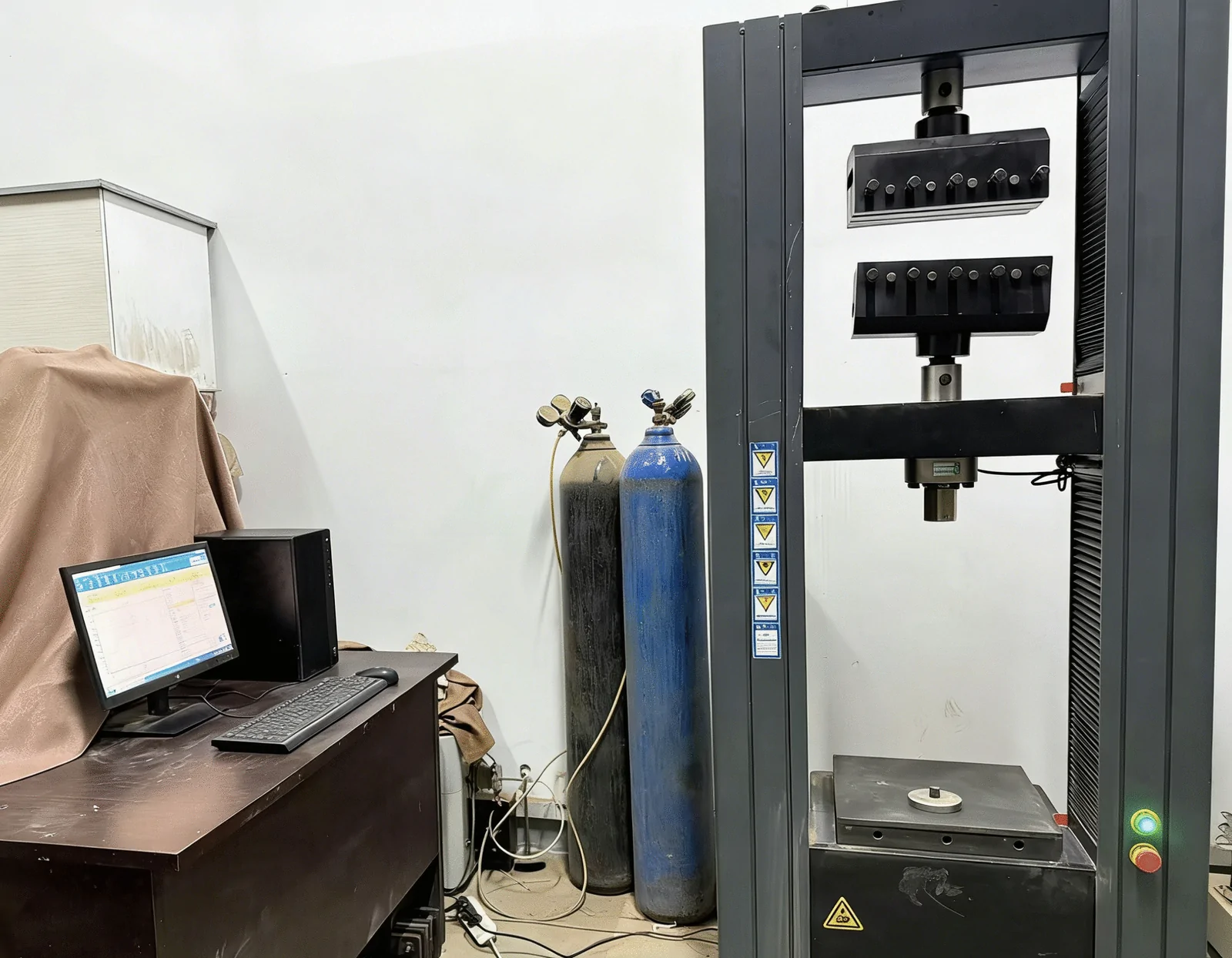
DW1210 universal tensile testing machine and full-width grips.

Customized mechanical grips clamped the full specimen width and maintained uniform contact during loading. A test was accepted when the specimen remained secure and failure occurred within the intended gauge region.

Specimens were prepared using a KST5123 sampling device equipped with customized cutting dies, as depicted in Fig 5. The integrated punching and depth-adjustment mechanisms enabled mechanized cutting, improved edge uniformity and dimensional accuracy, reduced operator-induced variability, and ensured consistency among tensile specimens.

**Fig 5:**
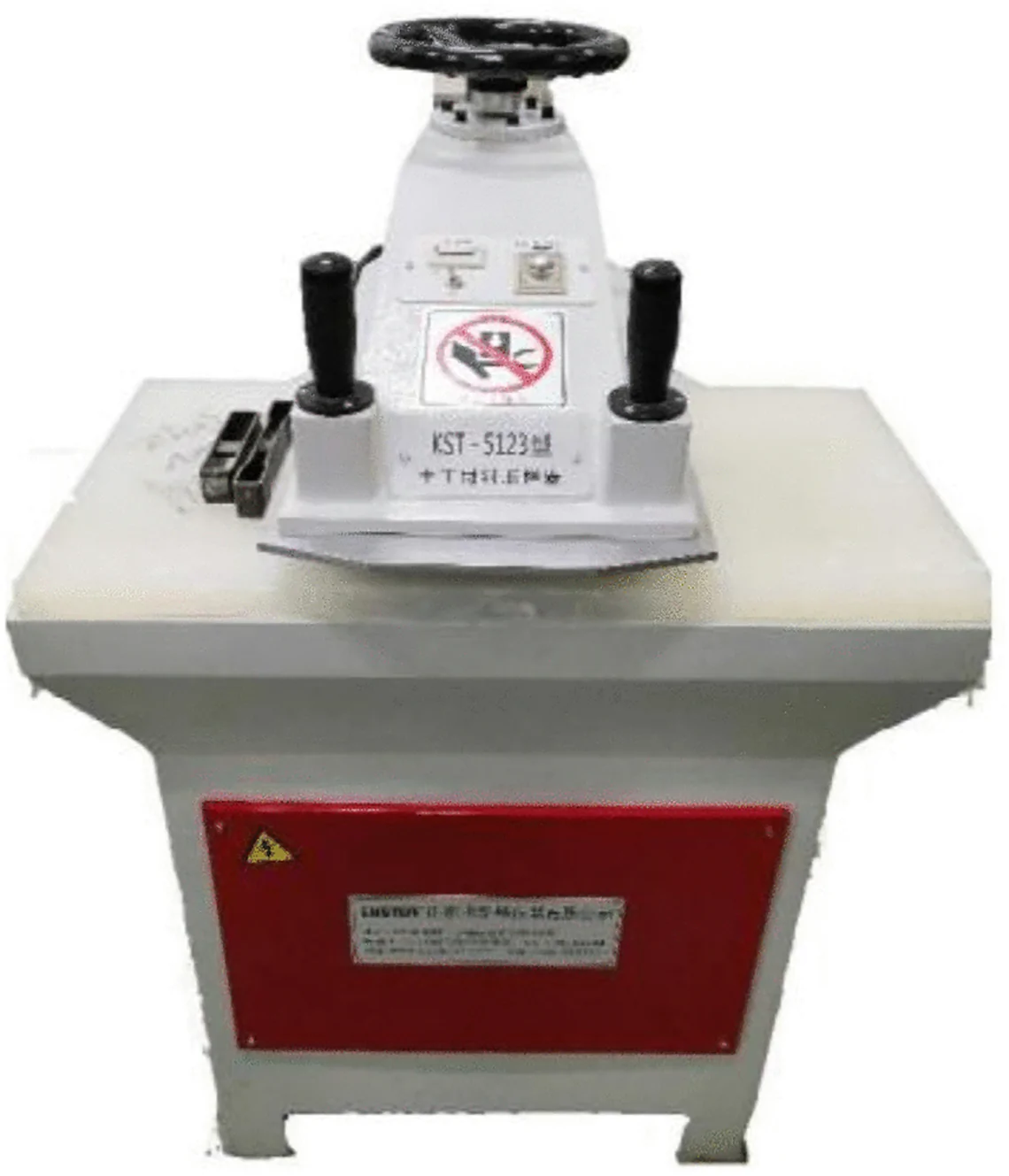
KST5123 sampling device used to prepare geocell specimens.

### 2.3 Testing procedures

The laboratory testing program was designed in accordance with GB/T 19274−2024, Geosynthetics—plastic geocells [24]. The selected configurations represent cell-wall tension, junction-inclusive cell-wall tension, junction seam, junction shear, and junction split. Fig 6 summarizes the specimen preparation and loading configurations. Specimen codes combine the geocell type (LW, IM, or UW), test subject (cell wall, junction-inclusive cell wall, or junction), and loading mode (tension, seam, shear, or split). The abbreviations are CW, JICW, J, TE, SE, SH, and SP, respectively.

**Fig 6:**
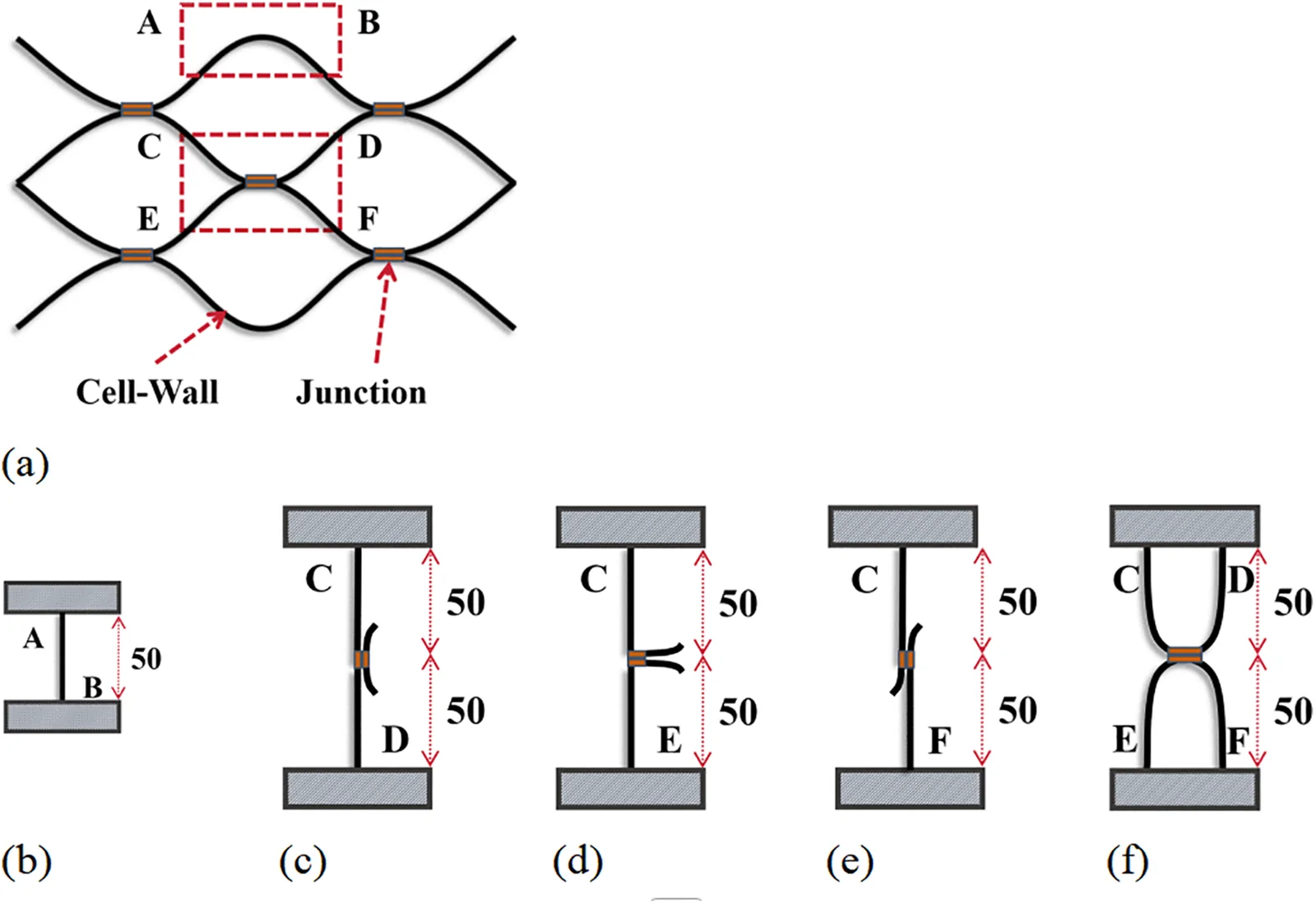
Specimen preparation and loading configurations. (a) sampling; (b) cell-wall tension; (c) junction-inclusive cell-wall tension; (d) junction seam; (e) junction shear; and (f) junction split. Dimensions are in mm.

The experimental program comprised three sequential phases designed to evaluate cell-wall tensile response and junction behavior.

(1) Cell-wall tensile tests established the tensile response of each product, as shown in Fig 6b.
(2) Junction-inclusive cell-wall tensile tests measured the response when load passed through the junction region, as shown in Fig 6c.
(3) Junction-specific seam, shear, and split tests were conducted to characterize junction resistance under distinct loading modes, as shown in Fig 6d-f.

All specimens were conditioned for at least 24 h at 23 ± 2 ℃ and 50% ± 10% relative humidity. All tests were conducted under the same environmental conditions. Specimens were cut along the longitudinal direction of the cell wall, with the junction centered in the gauge region for junction-inclusive cell-wall and junction tests. The crosshead speed was set at 50 mm/min in accordance with GB/T 19274−2024 [24]. Each test continued until specimen rupture. Results were discarded and the test was repeated when invalid failure modes, such as grip slippage or fracture at the clamp edge, were observed.

### 2.4 Statistical analysis

In accordance with the minimum sample requirement of GB/T 19274−2024 [24], five valid specimens were tested for each test group. Peak strength, strain at peak, and rupture strain were determined separately for each specimen. The arithmetic mean and sample standard deviation were then calculated from the specimen-level values. Mean strength–strain curves were obtained by averaging the recorded responses at corresponding acquisition points. The sample standard deviation of strength was calculated point by point. The solid lines in Figs 7, 10, 12, 14, and 16 represent the mean responses, and the shaded bands represent the mean ± 1 sample standard deviation (SD). The coefficient of variation was calculated as the sample standard deviation divided by the mean peak strength and was used to quantify the dispersion of the peak-strength results.

## 3 Results and discussion

### 3.1 Tensile behavior and fracture morphology of cell walls

Fig 7 shows the mean tensile strength–strain curves of the cell walls fabricated from the three polymers (PP, PET, and HDPE). The three cell-wall materials exhibited distinctly different mechanical responses. The LW cell wall (PP) showed an approximately linear increase in tensile strength without a distinct yield plateau. Its peak strength was 143.03 ± 4.43 kN/m at a strain of 14.26 ± 0.44%. The load-bearing capacity decreased rapidly after the peak, indicating brittle fracture. The IM cell wall (PET) exhibited an initial linear response and subsequent strain hardening. It reached the highest peak strength of 185.61 ± 7.96 kN/m at a strain of 30.48 ± 0.78%. Its tensile strength also decreased abruptly after the peak, reflecting high-modulus brittle failure. The UW cell wall (HDPE) displayed a clear yield stage and extensive plastic deformation. Its peak strength was 39.42 ± 2.41 kN/m at a strain of 63.24 ± 2.29%. A prolonged deformation stage followed the peak. Final rupture occurred at a strain of 235.24 ± 8.59%. LW and IM therefore showed relatively high strength and rapid post-peak failure. UW showed lower strength but substantially greater deformation capacity.

**Fig 7:**
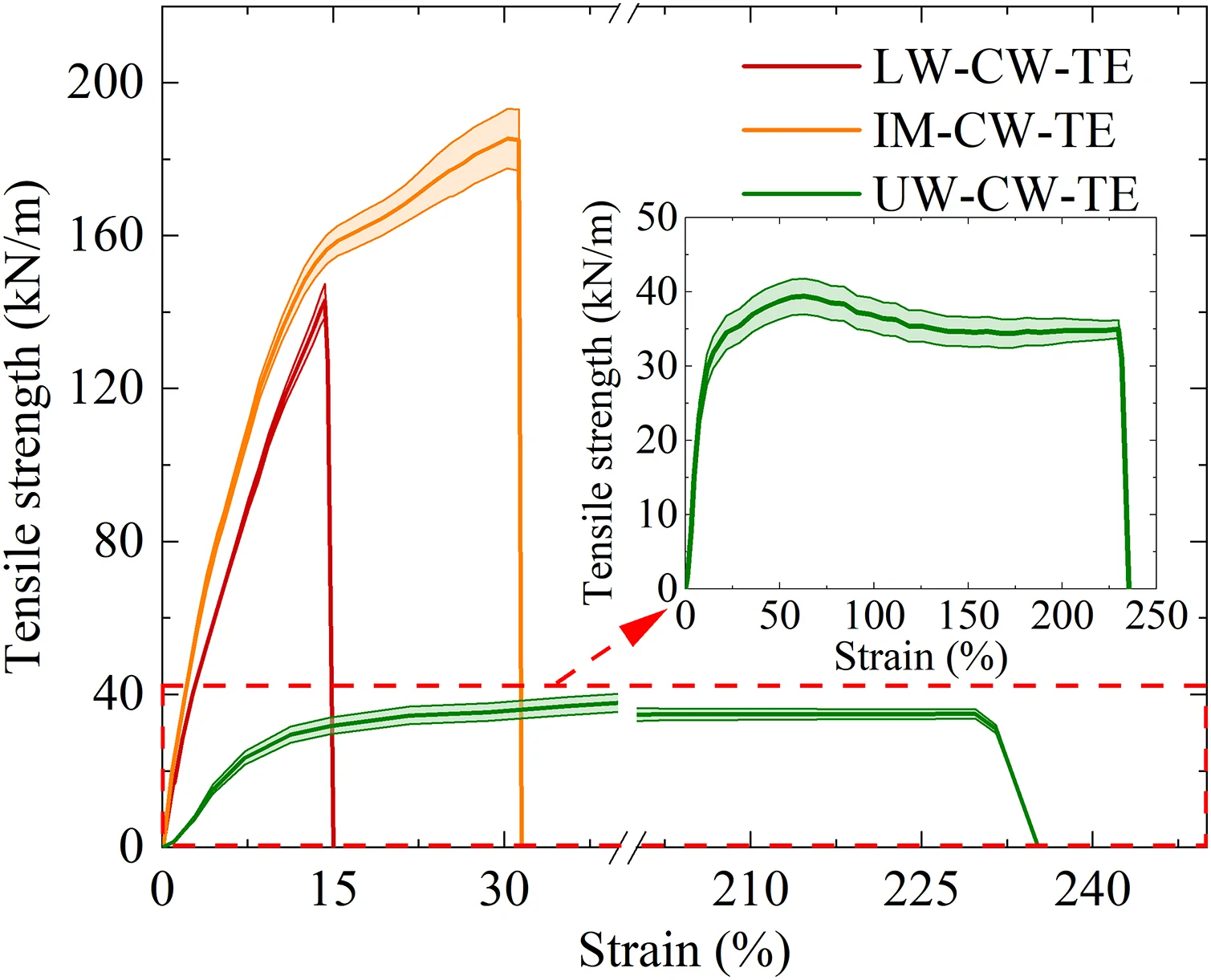
Mean tensile strength–strain curves of cell walls. Shaded bands show ±1 SD.

The macroscopic fracture patterns in Fig 8 agree with the curve shapes. The LW cell walls separated into fibrous filaments after rupture. Their fracture surfaces showed pronounced fibrillation. The IM cell walls showed no obvious necking. They formed flat fracture surfaces almost perpendicular to the loading direction, confirming brittle failure. By contrast, the UW cell walls underwent significant necking within the gauge section, accompanied by a shear lip oriented at approximately 45° to the loading direction in the necked region.

**Fig 8:**
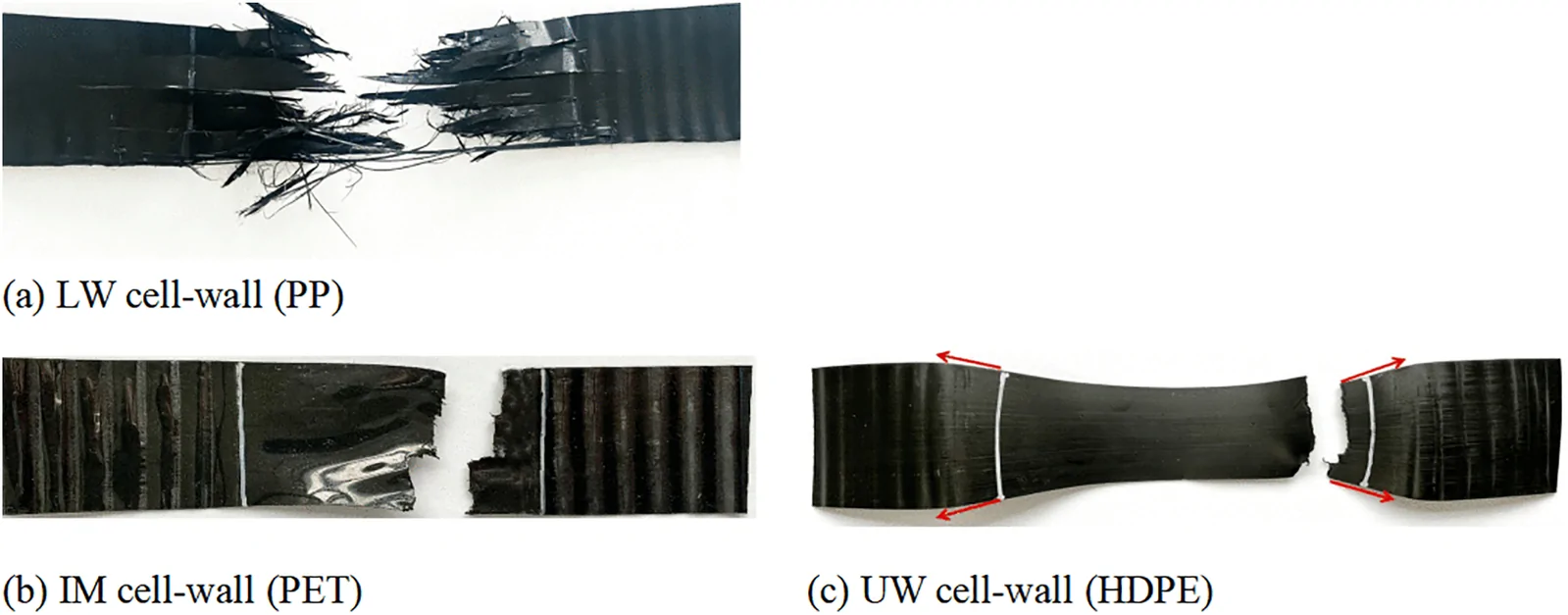
Macroscopic tensile failure patterns of cell walls. (a) LW; (b) IM; and (c) UW.

Fig 9 presents the SEM images of the three cell-wall tensile fracture surfaces at 1000 × magnification. The LW fracture surface contained both aligned and disordered microfibrillar bundles. This morphology corresponded to the filamentous separation observed at the macroscopic scale. The IM fracture surface was comparatively smooth and layered. Only limited fibrillation was observed. This morphology corresponded to the abrupt post-peak loss of tensile strength. The UW fracture surface was rough and densely covered with microfibrils of different lengths and diameters. These features reflected extensive plastic drawing and corresponded to the pronounced macroscopic necking.

**Fig 9:**
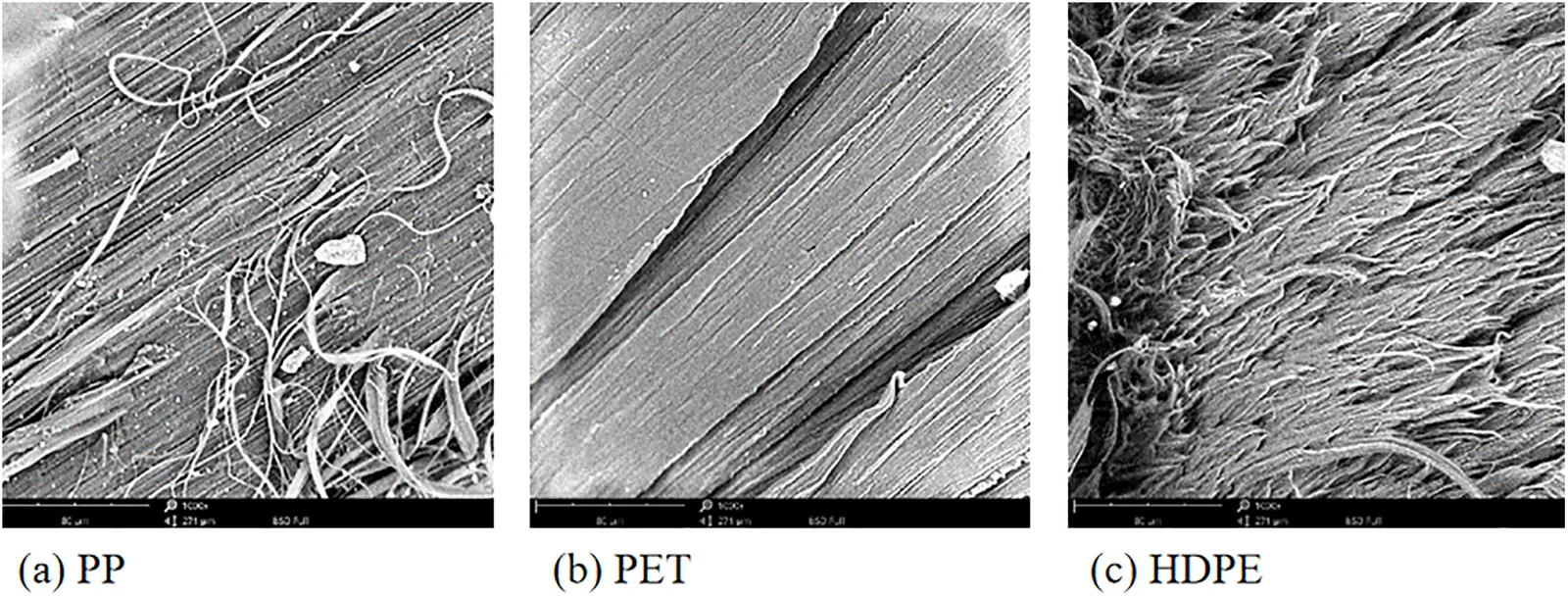
SEM images of cell-wall tensile fracture surfaces at 1000 × magnification. (a) PP; (b) PET; and (c) HDPE.

These differences in mechanical response and fracture morphology can be related to the polymer microstructure and geocell-forming process. The LW cell walls consisted of oriented PP. Tensile deformation promotes the unfolding and reorientation of microfibrillar crystallites. Cracks then propagate along the interfaces between adjacent microfibrils. Progressive microfibril pull-out, tearing, and rupture produce the observed filamentous morphology [25]. PET chains contain rigid benzene rings and flexible aliphatic groups; steric hindrance restricts chain rotation and imparts high rigidity [26]. After strain hardening, the limited chain mobility restricts stress redistribution. The IM cell walls therefore fractured abruptly. HDPE is a semicrystalline polymer. Initial deformation involves the rotation and rearrangement of chain segments in the amorphous regions. This process promotes yielding and plastic flow. Further deformation induces lamellar slip and molecular orientation. Drawn microfibrils consequently form along the loading direction. Microvoid nucleation, growth, and coalescence subsequently promote crack propagation and final rupture [27].

### 3.2 Junction-inclusive cell-wall tensile strength

Fig 10 shows the mean tensile strength–strain curves of junction-inclusive cell walls and the corresponding cell walls. All junction-inclusive specimens exhibited responses distinct from those of the corresponding cell walls. The LW junction-inclusive cell walls showed an approximately linear increase in tensile strength without a distinct yield point. After the peak, the tensile strength rapidly decreased to zero. The specimens reached a peak strength of 184.50 ± 6.76 kN/m at a strain of 16.92 ± 0.53%. Compared with the corresponding LW cell walls, the peak tensile strength and peak strain increased by 28.99% and 18.63%, respectively. The IM specimens also showed an approximately linear strength increase, but no strain-hardening stage was observed. They reached a peak strength of 74.63 ± 4.53 kN/m at a strain of 13.24 ± 0.46%. Compared with the corresponding IM cell walls, the peak tensile strength and peak strain decreased by 59.79% and 56.55%, respectively. The UW specimens followed a mechanical evolution similar to that of the corresponding UW cell walls. The response included linear elasticity, yielding, plastic deformation, and final rupture. The specimens reached a peak strength of 30.05 ± 2.71 kN/m at a strain of 36.85 ± 5.81%. Compared with the corresponding UW cell walls, the peak tensile strength and peak strain decreased by 23.76% and 41.73%, respectively.

**Fig 10:**
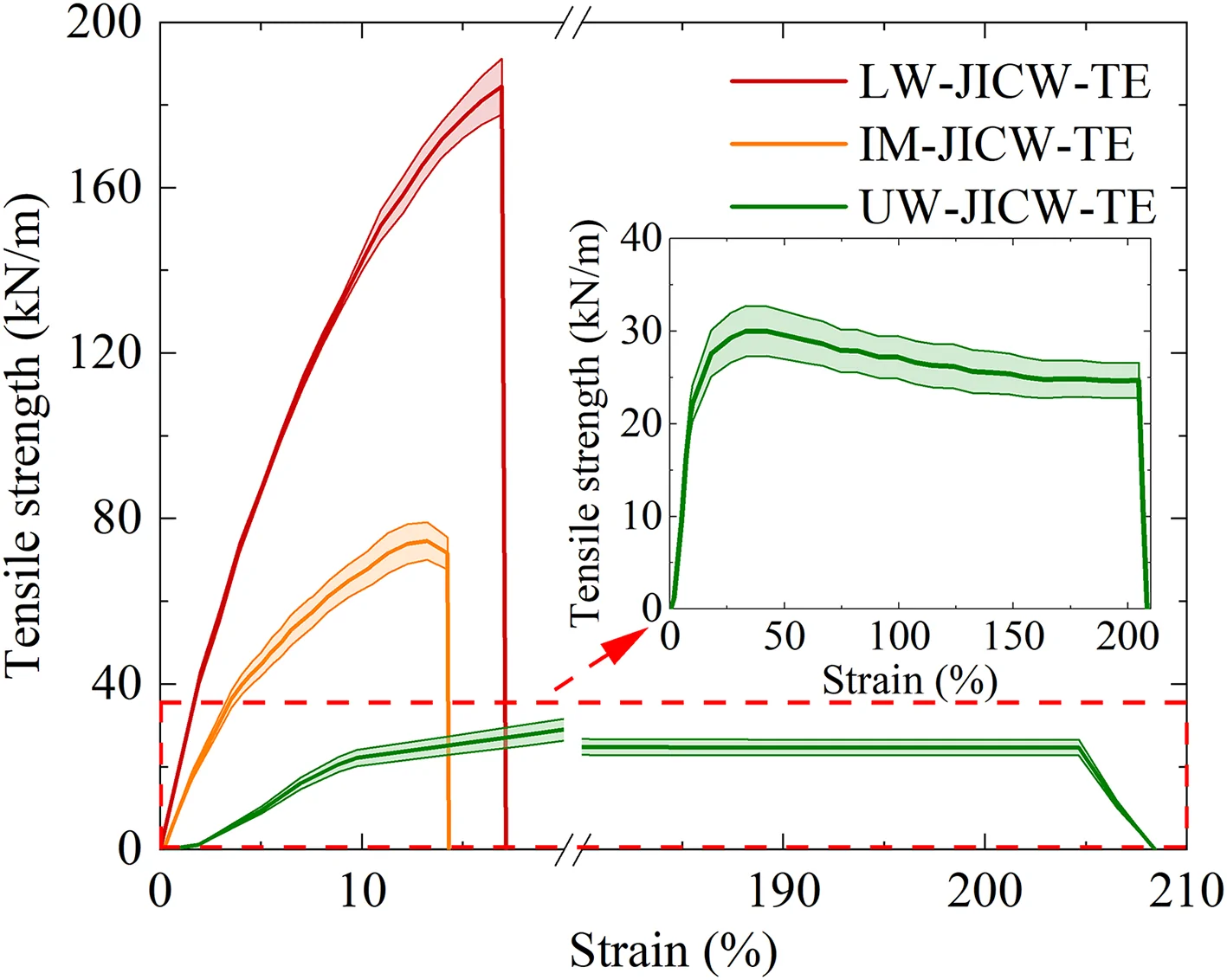
Mean tensile strength–strain curves of junction-inclusive cell walls. Shaded bands show ±1 SD.

Fig 11 shows the macroscopic failure characteristics of the three junction-inclusive cell-wall types. In all three geocell products, failure was concentrated at or adjacent to the junction region. The specimens differed in local deformation, crack path, and final failure position. The LW specimens exhibited strip-like tearing near the raised-tab edge. The raised tab and heat-fused interface remained intact after failure. The integral raised tab preserved cell-wall continuity because its formation required neither perforation nor cutting. Its larger local section transferred the tensile load toward the tab–wall transition, where the abrupt change in thickness concentrated deformation. Tearing initiated at this transition and propagated into the cell wall, while the raised tab and heat-fused interface remained intact.

**Fig 11:**
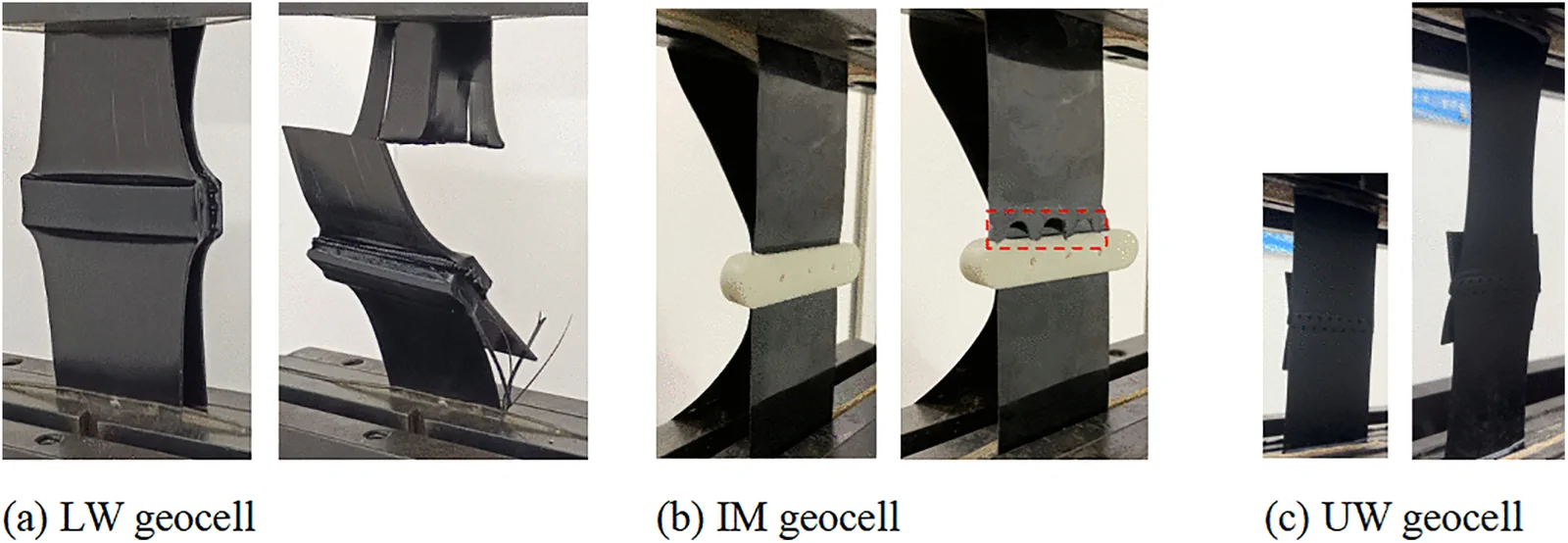
Macroscopic tensile failure patterns of junction-inclusive cell walls. (a) LW; (b) IM; and (c) UW.

For the IM junctions, the pre-formed holes interrupted cell-wall continuity and reduced the net load-bearing section. Tensile stress was diverted around the holes and concentrated at their edges and in the intervening ligaments. Although the injection-molded body transferred load through the hole walls, it did not restore the continuity of the PET strip. During loading, the holes elongated into ellipses, and cracks propagated from their edges through the ligaments to form the final failure path. The reduced net section and deformation concentration around the holes were associated with the lower peak strength and strain at peak of the IM junction-inclusive specimens. The UW specimens underwent pronounced necking within the gauge length. The welded overlap showed less deformation than the adjacent HDPE walls. This deformation difference concentrated strain at the two weld–wall boundaries. Two symmetric necking zones therefore developed on both sides of the weld, and final fracture occurred at one of these boundaries. The reductions in peak strength and strain at peak were accompanied by strain localization at the two weld–wall boundaries.

### 3.3 Junction seam strength

Fig 12 presents the mean seam strength–strain curves of the junctions of the three geocell products under seam loading. The LW, IM, and UW specimens exhibited distinct mechanical responses. The LW junctions showed a continuous increase in seam strength with strain and entered a strain-hardening stage after approximately 10% strain. The LW curve reached a peak of 66.16 ± 4.20 kN/m at 14.06 ± 1.08% strain, followed by sudden fracture. This response combined the highest seam strength with abrupt post-peak failure. The IM junctions rapidly increased in strength to a peak of 52.21 ± 4.49 kN/m at 5.90 ± 0.21% strain and then dropped sharply to zero at 7.08 ± 0.27% strain. The sharp load drop and early complete separation indicated relatively high seam strength but limited deformation capacity. The UW junctions gradually increased to a peak of 10.47 ± 0.50 kN/m at 5.38 ± 0.24% strain, followed by progressive strength degradation to zero at 14.63 ± 0.60% strain. The UW junctions therefore showed the lowest seam strength but a more gradual post-peak failure process. The three geocell products exhibited different seam capacities and post-peak responses.

**Fig 12:**
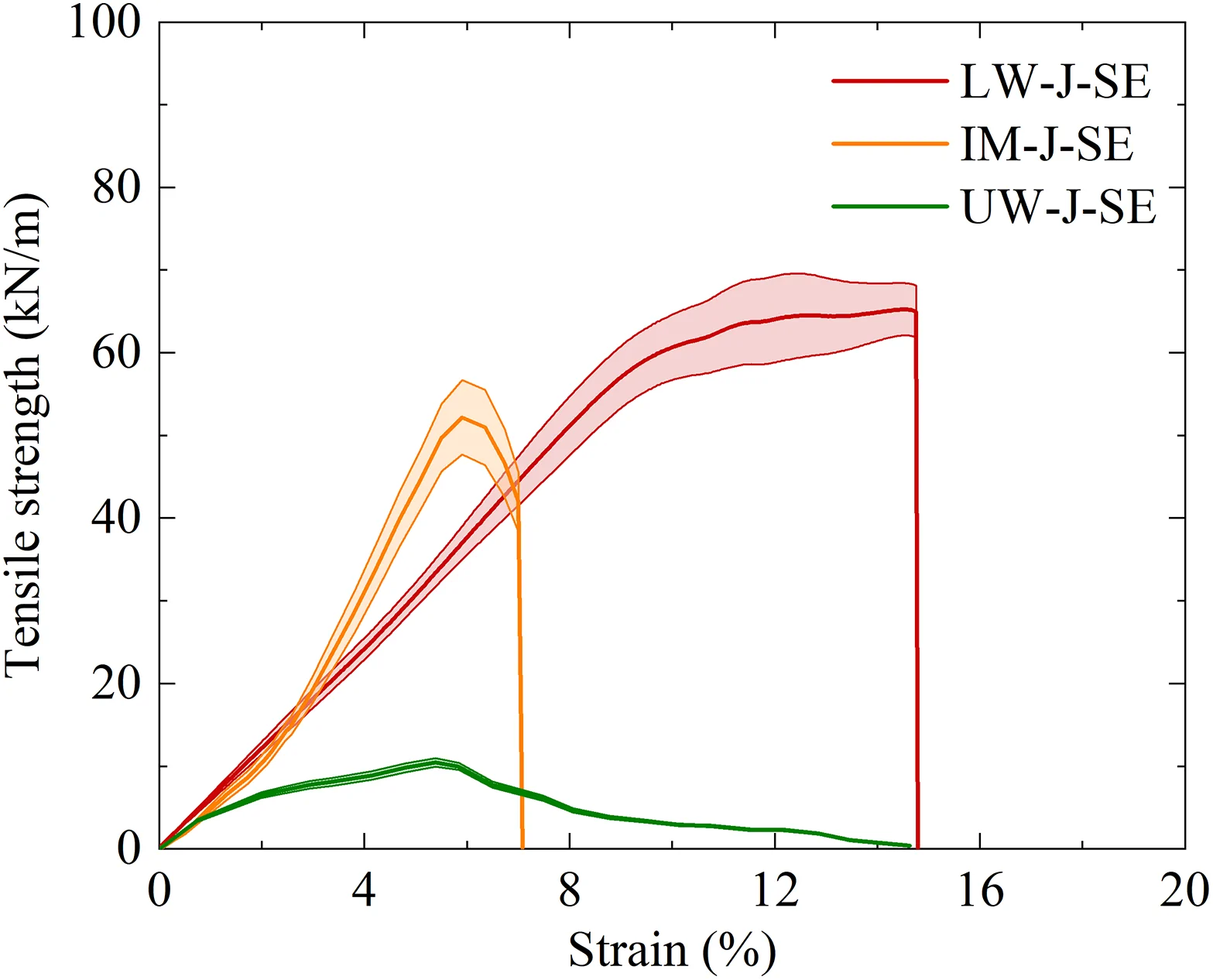
Mean junction seam strength–strain curves. Shaded bands show ±1 SD.

Fig 13 shows the macroscopic failure characteristics under seam loading. The LW and IM specimens fractured abruptly, whereas the UW specimens failed after progressive load loss. No obvious necking was observed in any seam specimen. Failure initiated at the cell-wall connection regions adjacent to the junctions. The LW cell walls tore from the edge of the raised tab. The LW specimen showed a comparatively flat macroscopic fracture surface with limited filamentous separation. The IM junctions fractured at the edges of the pre-formed perforations. The perforations reduced the continuous load-bearing section, and cracks initiated at their edges before propagating through the intervening wall sections. The UW junctions failed within the weld zone after a gradual reduction in load-bearing capacity. These failure paths corresponded to the local construction of each junction. Seam loading transferred tensile force through the raised-tab edge in LW, the perforated wall section in IM, and the welded connection zone in UW.

**Fig 13:**
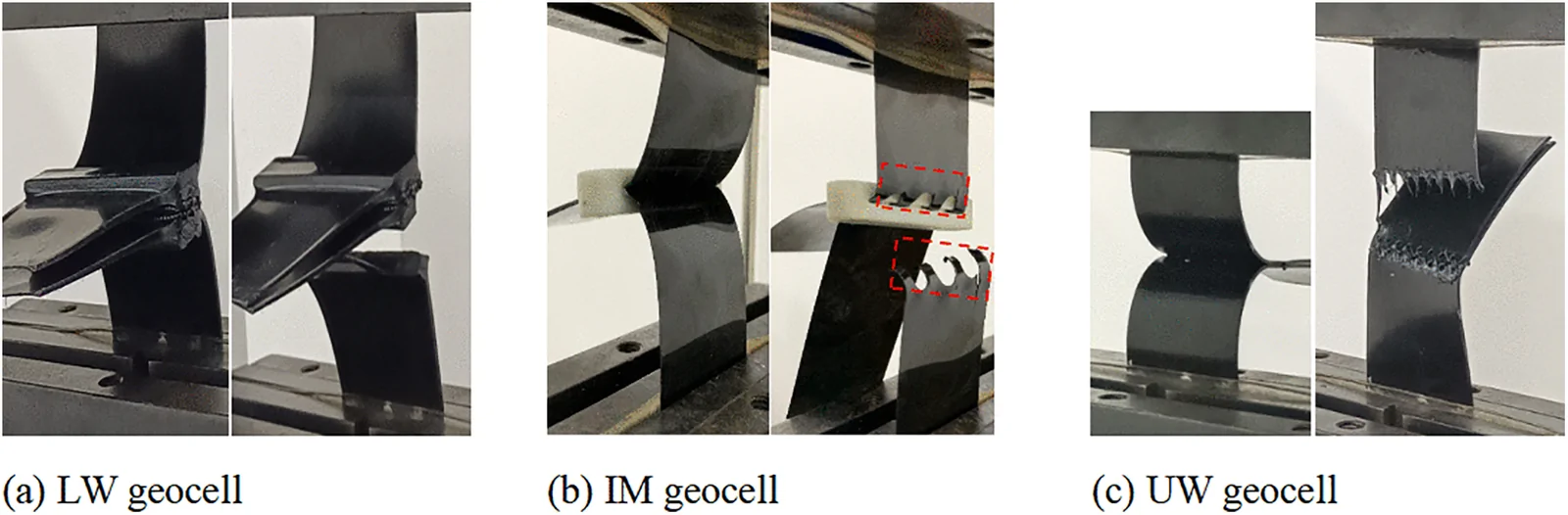
Macroscopic junction failure patterns under seam loading. (a) LW; (b) IM; and (c) UW.

### 3.4 Junction shear strength

Fig 14 presents the mean shear strength–strain curves of the junctions of the three geocell products. The LW junctions showed an approximately linear increase in strength with strain and reached a peak of 146.02 ± 8.46 kN/m at 13.43 ± 0.44% strain before sudden fracture. This response represented the highest shear capacity and an abrupt post-peak failure process. Both the IM and UW junctions initially exhibited linear strength increases. The IM junctions reached a peak strength of 31.03 ± 1.39 kN/m at 3.35 ± 0.28% strain, followed by yielding and strain softening. Final failure occurred at 13.88 ± 0.54% strain. The UW junctions developed an early peak of approximately 20 kN/m, followed by yielding and strain softening. Unlike the IM specimens, the UW junctions subsequently regained strength gradually during an extended deformation stage. Their maximum strength was 22.63 ± 2.26 kN/m. Final rupture occurred at 343.09 ± 13.72% strain. During the extended post-yield stage, the HDPE wall segments rotated, stretched, and progressively aligned with the loading direction. This deformation of the wall segments produced the large rupture strain recorded under shear loading.

**Fig 14:**
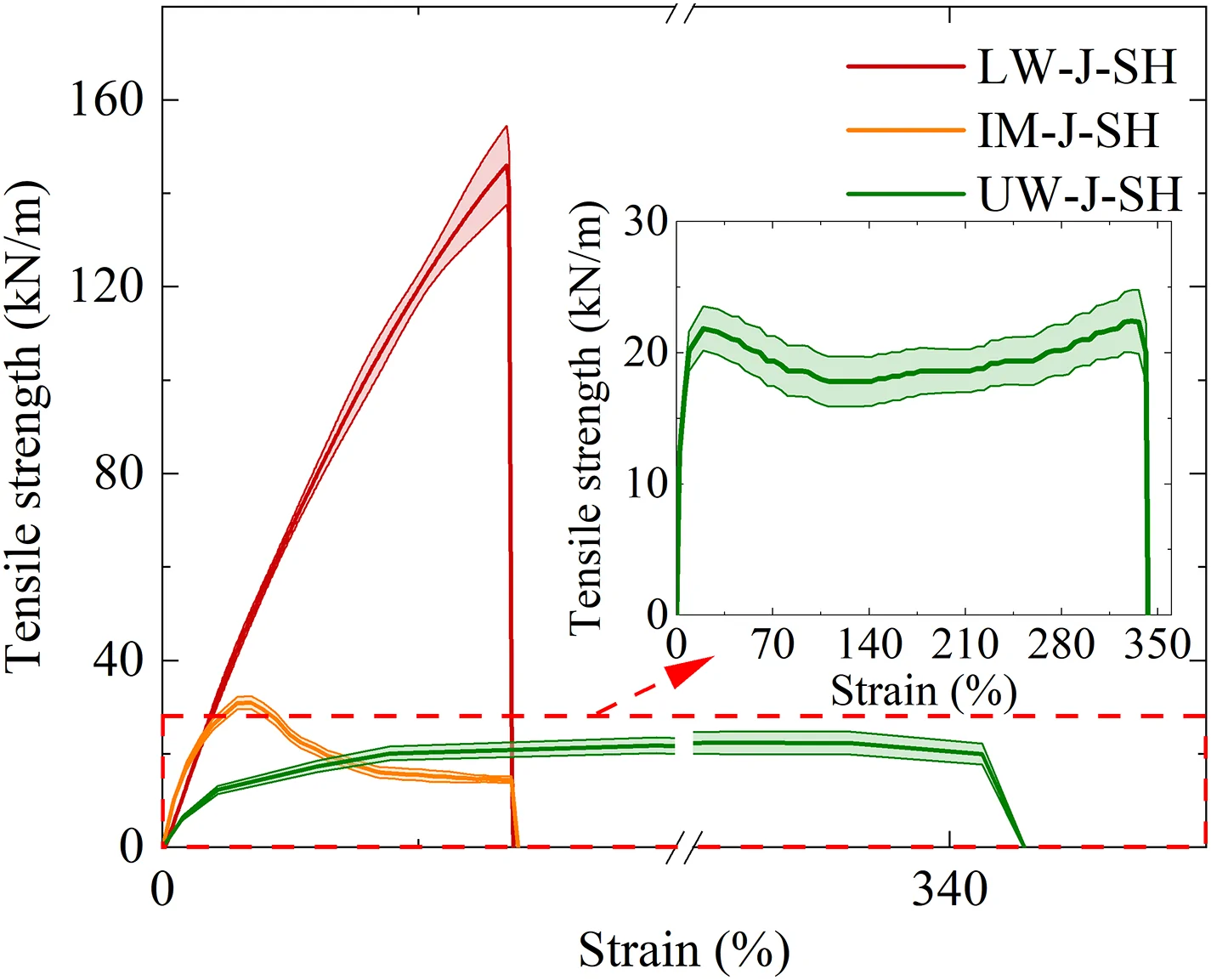
Mean junction shear strength–strain curves. Shaded bands show ±1 SD.

Fig 15 shows the macroscopic failure characteristics under shear loading. The macroscopic failure patterns were similar to those observed in the junction-inclusive tensile tests described in Section 3.2. The LW junctions fractured near the tab–wall transition. The fracture surface was relatively flat and showed strip-like tearing of the cell walls. The IM junctions fractured at the perforations. Under load, the circular holes elongated into ellipses. Under shear loading, the junction constraint created an asymmetric local load-transfer path. Deformation concentrated at the hole wall farthest from the loading direction. This localized deformation increased stress concentration and produced visible warping before fracture. The UW junctions showed deformation and fracture patterns similar to those observed under junction-inclusive tension. Two necking zones formed on opposite sides of the weld. Final fracture occurred at a weld–wall boundary after large deformation.

**Fig 15:**
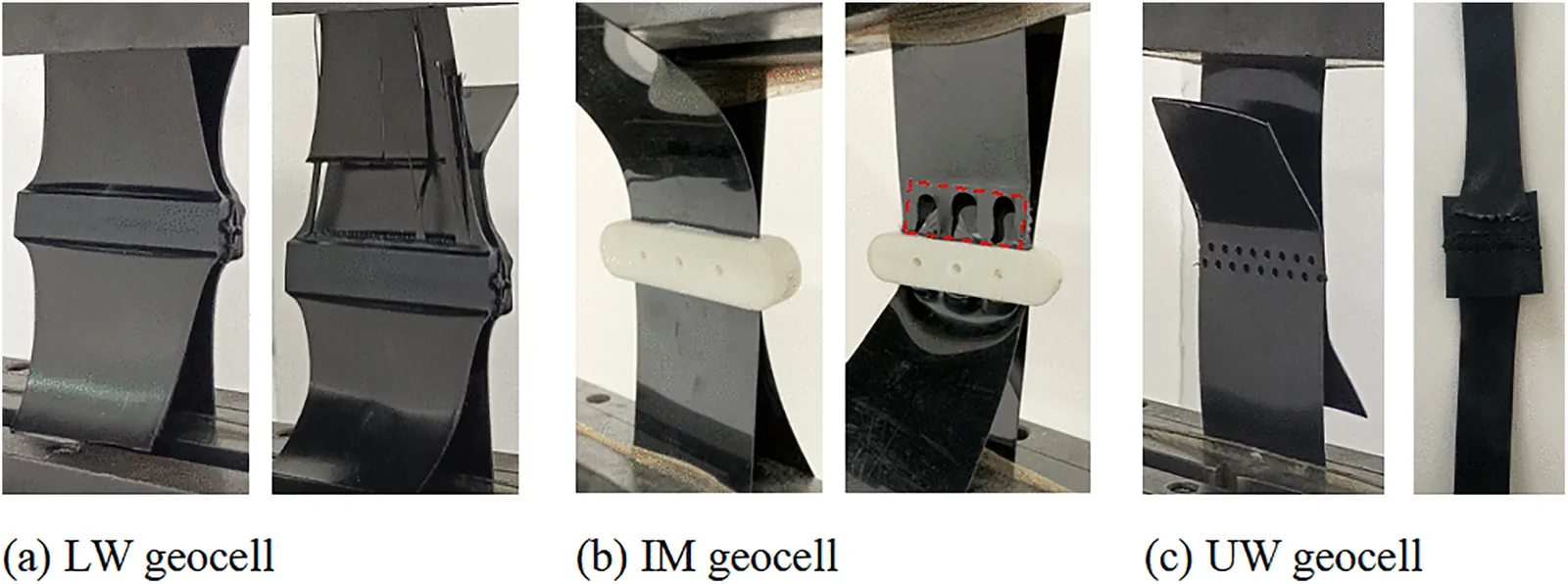
Macroscopic junction failure patterns under shear loading. (a) LW; (b) IM; and (c) UW.

### 3.5 Junction split strength

Fig 16 presents the mean split strength–strain curves of the junctions of the three geocell products. The LW junctions showed an approximately linear increase in split strength. They reached a peak strength of 113.30 ± 6.08 kN/m at 20.69 ± 0.68% strain. Abrupt fracture followed the peak, and no obvious plastic deformation was observed. The IM junctions showed a similar trend but higher strength and lower strain at peak. They reached a peak split strength of 158.55 ± 10.34 kN/m at 5.42 ± 0.20% strain before a sharp strength drop. The IM junctions therefore provided the highest split strength but lost their load-carrying capacity immediately after the peak. Conversely, the UW junctions exhibited clear elastoplastic softening. Their strength developed slowly during the initial loading stage and reached a lower peak of 29.46 ± 2.70 kN/m at 22.27 ± 0.85% strain. Rather than failing immediately after the peak, the specimens entered a continuous plastic-softening stage. The split strength decreased gradually with increasing deformation. Load-carrying capacity was completely lost only when the strain reached 31.60 ± 1.25%. Among the three junction loading modes, seam loading produced the lowest peak strength for LW and UW, whereas shear loading produced the lowest peak strength for IM. Across the 15 test groups, the coefficients of variation of peak strength ranged from 3.10% to 9.98%.

**Fig 16:**
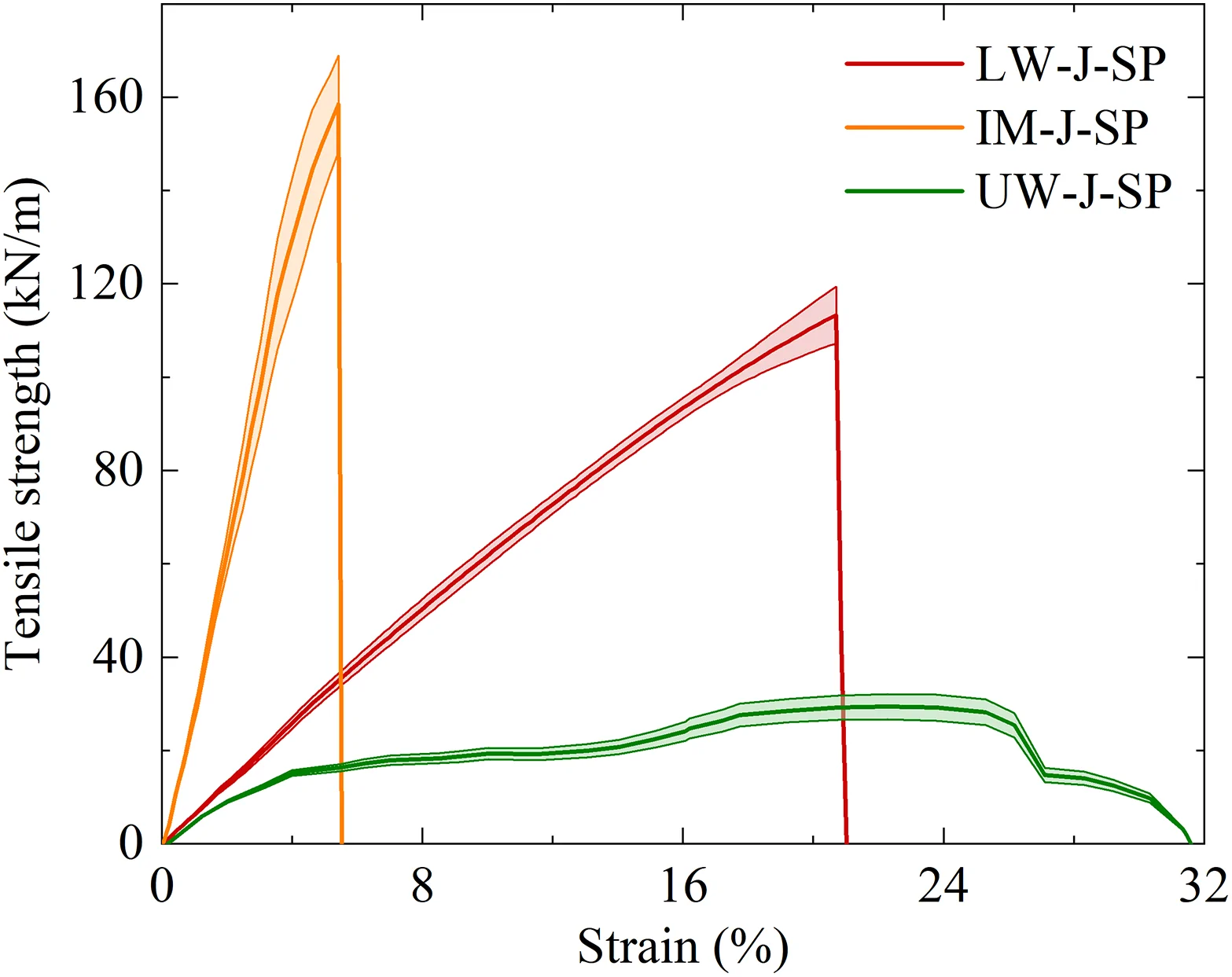
Mean junction split strength–strain curves. Shaded bands show ±1 SD.

Fig 17 shows the macroscopic failure characteristics under split loading. The failure positions differed from those observed under seam and shear loading. Both LW and IM junctions fractured abruptly. The LW junctions separated at the heat-fused interface, producing a relatively flat fracture surface with no obvious fibrillation. The IM junctions failed through fracture of the injection-molded junction body. In contrast, the UW junctions separated through the central weld region after visible plastic tensile deformation.

**Fig 17:**
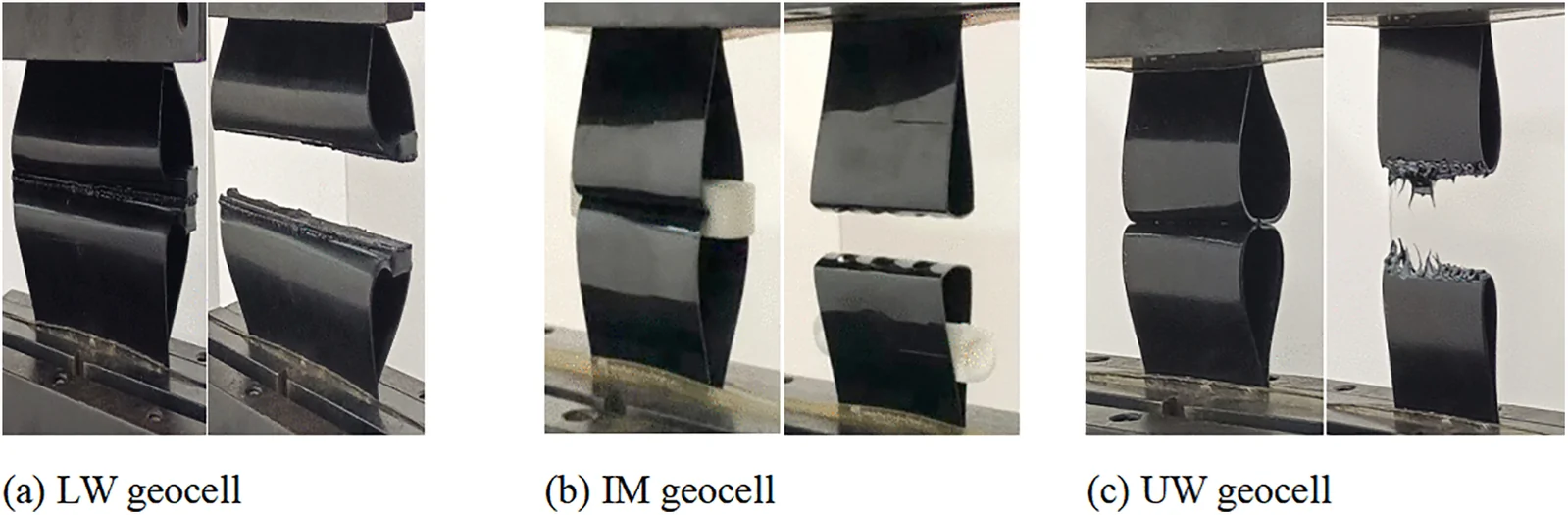
Macroscopic junction failure patterns under split loading. (a) LW; (b) IM; and (c) UW.

## 4 Conclusion

This study compared the tensile behavior and failure processes of LW, IM, and UW commercial geocells through laboratory tensile and junction-specific tests. The testing program included cell-wall tensile tests, junction-inclusive cell-wall tensile tests, and junction seam, shear, and split tests. The main conclusions are as follows:

(1) The LW and IM cell walls reached peak strengths of 143.03 ± 4.43 and 185.61 ± 7.96 kN/m, respectively. Both types lost load rapidly after the peak. The LW cell walls separated into filamentous strands. The IM cell walls formed comparatively flat fracture surfaces. In contrast, the UW cell walls showed yielding, pronounced necking, and extended plastic deformation. Their peak strength was 39.42 ± 2.41 kN/m, and final rupture occurred at 235.24 ± 8.59% strain. SEM observations showed microfibrillar bundles in LW, a comparatively smooth layered surface in IM, and dense drawn microfibrils in UW.
(2) Including the junction region in the tensile load path markedly changed the tensile response relative to the corresponding cell wall. The peak strength of the LW junction-inclusive specimens was 28.99% higher than that of the LW cell walls, and tearing occurred near the raised-tab edge. The corresponding peak strengths of the IM and UW specimens were 59.79% and 23.76% lower, respectively. IM specimens fractured through the ligaments between the pre-formed holes, whereas UW specimens fractured at a weld–wall boundary after symmetric necking.
(3) Among the tested commercial geocells, LW provided the highest seam and shear strengths, reaching 66.16 ± 4.20 and 146.02 ± 8.46 kN/m, respectively. IM provided the highest split strength of 158.55 ± 10.34 kN/m but reached the peak at a strain of only 5.42 ± 0.20%. UW showed lower peak junction strengths but the largest deformation under shear loading. Progressive rotation and stretching of the HDPE wall segments resulted in a rupture strain of 343.09 ± 13.72%.
(4) The three commercial geocells showed distinct failure paths under the investigated junction loading modes. LW junctions failed near the tab–wall transition under seam and shear loading and at the heat-fused interface under split loading. IM junctions failed at the hole edges under seam and shear loading and through the injection-molded junction body under split loading. UW junctions failed within the weld region under seam loading, at a weld–wall boundary under shear loading, and through the central weld under split loading.

## Supporting information

S1 Data Underlying numerical data for the figures and statistical analyses. (XLSX)
